# Identification of a PEST Sequence in Vertebrate K_IR_2.1 That Modifies Rectification

**DOI:** 10.3389/fphys.2019.00863

**Published:** 2019-07-05

**Authors:** Muge Qile, Yuan Ji, Marien J. C. Houtman, Marlieke Veldhuis, Fee Romunde, Bart Kok, Marcel A. G. van der Heyden

**Affiliations:** Department of Medical Physiology, Division of Heart and Lungs, University Medical Center Utrecht, Utrecht, Netherlands

**Keywords:** K_IR_2.1, inward rectifier, PEST domain, vertebrates, patch clamp, potassium, channel

## Abstract

K_IR_2.1 potassium channels, producing inward rectifier potassium current (*I*_*K1*_), are important for final action potential repolarization and a stable resting membrane potential in excitable cells like cardiomyocytes. Abnormal K_IR_2.1 function, either decreased or increased, associates with diseases such as Andersen-Tawil syndrome, long and short QT syndromes. K_IR_2.1 ion channel protein trafficking and subcellular anchoring depends on intrinsic specific short amino acid sequences. We hypothesized that combining an evolutionary based sequence comparison and bioinformatics will identify new functional domains within the C-terminus of the K_IR_2.1 protein, which function could be determined by mutation analysis. We determined PEST domain signatures, rich in proline (P), glutamic acid (E), serine (S), and threonine (T), within K_IR_2.1 sequences using the “epestfind” webtool. WT and ΔPEST K_IR_2.1 channels were expressed in HEK293T and COS-7 cells. Patch-clamp electrophysiology measurements were performed in the inside-out mode on excised membrane patches and the whole cell mode using AxonPatch 200B amplifiers. K_IR_2.1 protein expression levels were determined by western blot analysis. Immunofluorescence microscopy was used to determine K_IR_2.1 subcellular localization. An evolutionary conserved PEST domain was identified in the C-terminus of the K_IR_2.1 channel protein displaying positive PEST scores in vertebrates ranging from fish to human. No similar PEST domain was detected in K_IR_2.2, K_IR_2.3, and K_IR_2.6 proteins. Deletion of the PEST domain in California kingsnake and human K_IR_2.1 proteins (ΔPEST), did not affect plasma membrane localization. Co-expression of WT and ΔPEST K_IR_2.1 proteins resulted in heterotetrameric channel formation. Deletion of the PEST domain did not increase protein stability in cycloheximide assays [T½ from 2.64 h (WT) to 1.67 h (ΔPEST), n.s.]. WT and ΔPEST channels, either from human or snake, produced typical *I*_*K1*_, however, human ΔPEST channels displayed stronger intrinsic rectification. The current observations suggest that the PEST sequence of K_IR_2.1 is not associated with rapid protein degradation, and has a role in the rectification behavior of *I*_*K1*_ channels.

## Introduction

Since its cloning in the early 1990s (Kubo et al., 1993), many domains of the K_IR_2.1 primary protein sequence, encoded by *KCNJ2*, have been attributed to biological function and molecular structure, but not all. K_IR_2.1 expression is found in a variety of excitable and non-excitable cells, like skeletal, smooth and cardiac muscle cells, neuronal cells, juxtaglomerular, and endothelial cells (De Boer et al., 2010). The resulting inward rectifying potassium current (*I*_*K1*_) is characterized by a larger inward than outward current from equal negative and positive deflections from the potassium equilibrium potential. This property allows for action potential formation in excitable cells, while providing a stable resting membrane potential in between action potentials (Van der Heyden and Jespersen, 2016). K_IR_2.1 carried potassium inward rectifying channels are tetramers of four K_IR_2.1 subunits. Other K_IR_2.x isoforms may form homotetramers also, and some can combine with K_IR_2.1 to form heterotetramers with slightly altered conductive characteristics compared to their respective homotetramers (e.g., Preisig-Müller et al., 2002). Mutations in the K_IR_2.1 gene associate with Andersen-Tawil Syndrome and congenital atrial fibrillation in patients. Therefore, more understanding of the functions of different protein domains might provide new avenues for therapeutic approaches.

Several discrete domains within the K_IR_2.1 sequence have been associated with certain functions, like potassium selectivity [amino acid (aa) 144-146], Endoplasmic Reticulum (ER) export (aa 374-379; Ma et al., 2001; Stockklausner et al., 2001), Golgi export (aa 44-61 and 314-322; Hofherr et al., 2005; Ma et al., 2011), a PDZ binding domain (aa 425-427, Leonoudakis et al., 2004), a Caveolin3 binding motif (aa 81-88; Vaidyanathan et al., 2018). K_IR_2.1 and K_IR_2.2 crystal structure and homology modeling provided additional 3-dimensional information and showed a K_IR_2.1 channel containing a transmembrane pore domain with a long intracellular pore extension formed by the so-called cytoplasmic pore domain (Pegan et al., 2005; Hansen et al., 2011; Lee et al., 2013). Furthermore, the structures provided compelling mechanistic insights into essential residues/domains involved in rectification (Tao et al., 2009). Three amino acids (D172, E224, and E299) in the pore regions are essential for rectification, i.e., reducing outward potassium flow upon depolarization. D172 is located in the transmembrane domain and is involved in so-called deep pore polyamine and Mg^2+^ binding, whereas E224 and E299 are located in the cytoplasmic pore domain and also bind polyamines and Mg^2+^.

PEST domains are regions rich in proline (P), glutamic acid (E), aspartic acid (D), serine (S), and threonine (T) confined by two positively charged amino acids, lysine (K), arginine (R) or histidine (H). These domains were first identified in short living proteins and the PEST domain function was therefore deduced as protein instability domains (Rogers et al., 1986). Indeed, in many short living proteins, mutation of the PEST domain resulted in stabilization of the protein (Rechsteiner and Rogers, 1996). Furthermore, in a number of proteins PEST domains appeared to function as anchor site of E3 ubiquitin ligases (Xing et al., 2010; Meyer et al., 2011; Li et al., 2018) required for, but not limited to, ubiquitin dependent protein degradation. However, specific deletion of PEST domains did not always increase protein half life (Nixon et al., 1995), PEST domains were found also in many long-lived proteins and additional or alternative functions have been attributed to PEST domains, like intracellular sorting, binding of the SUMO conjugating protein Ubc9 or binding of the second plastoquinone electron acceptor (Nixon et al., 1995; Bies et al., 2002; Zhuang et al., 2012). Upon cloning and aligning of a large number of K_IR_2.1 protein sequences (Houtman et al., 2014) we noticed an amino acid stretch that might fulfill the criteria of a PEST domain. We hypothesized that K_IR_2.1 proteins contain a PEST domain in their C-terminus and set out to determine its biological function.

## Materials and Methods

### PEST Domain Identification

Protein sequences were individually loaded in the EMBOSS program ePESTfind tool^1^ using the standard settings.

### Mutations

Human *Hs*K_IR_2.1ΔPEST was constructed by PCR amplification of a part of *HsKCNJ2* (Jansen et al., 2008) from pGEM-T-easy using T7 forward and a specifically designed reverse primer (CAGTCATATCTCCGACTCTCGCCGTAAGGGCCTGGGCTCTAGAGGTACACTTGCCTGGTTGCTTGTGAGGGCAACTTC). The amplification product contained the entire human *KCNJ2* open reading frame sequence with an in-frame deletion of the complete PEST sequence (KEEDDSENGVPE STSTDTPPDIDLH) and was cloned in pGEM-T-easy and subsequently subcloned into pcDNA4 (Life-Technologies). The similar procedure was followed for constructing California kingsnake *Lg*K_IR_2.1ΔPEST using *LgKCNJ2* (Houtman et al., 2014) and the designed reverse primer (CAGAGTCATATTTCAGATTCTCGCCTTAAAGGTCTTGGTTCTAGGGGCACCCCTGCTTGGCTAAGATGGTCCATCTCTGGGCCCGCAAGGGCAACTTC) that resulted in deletion of the complete snake K_IR_2.1 PEST sequence (KEEEDSDNGVPESTSTDTH).

### Cell Culture

HEK293T and COS-7 cells were cultured in Dulbecco’s Modified Eagles Medium (DMEM; Lonza, Breda, Netherlands) supplemented with 10% fetal calf serum (FCS; Sigma-Aldrich, Zwijndrecht, Netherlands), 2 mM L-glutamine (Lonza), and 50 U/mL penicillin and 50 mg/mL streptomycin (both Lonza) at 37°C with 5% CO_2_. In time course experiments, cells for each time point were seeded on the same day, and drugs were added for the indicated time prior to harvest of all samples. For patch clamp electrophysiology, 3 days prior to measurements, HEK293T cells were grown on poly-L-lysine (Sigma-Aldrich) coated Ø 12 mm cover slips and transfected with human K_IR_2.1 (WT or ΔPEST) using Lipofectamine 2000 (Invitrogen, Breda, Netherlands) according to the manufacturer’s protocol. Recordings were performed 24 h after transfection. In western blot experiments, HEK293T cells were grown on 60 mm tissue culture dishes and transfected using linear PEI as described earlier (Ji et al., 2017a). In immunofluorescence microscopy experiments, COS-7 cells were grown on Ø 15 mm coverslips, pre-coated with poly-L-lysine (Sigma-Aldrich), and transfected with K_IR_2.1 (WT or ΔPEST) using Lipofectamine according to the manufacturer’s protocol.

### Drugs

Chloroquine (Sigma, St. Louis, MO, United States, cat. No. C6628) was dissolved in sterile water at a concentration of 10 mM and stored at −20°C. Cycloheximide (Sigma, cat. No. C7698) was dissolved in ethanol at a concentration of 5 mg/mL, stored and aliquoted at −20°C until use. SPM was prepared in DEPC water at a concentration of 50 mM. All drugs were diluted on the day used.

### Immunohistochemistry and Fluorescence Microscopy

COS-7 cells were stained essentially as described earlier (Ji et al., 2017a). Antibodies used were K_IR_2.1 (1:250; Santa Cruz Biotechnology, Heidelberg, Germany, cat. no. sc-18708), Pan-Cadherin (1:800, Sigma-Aldrich, St. Louis MO, United States, cat. no. C1821). Cell nuclei were stained with 4′,6-diamidino-2-phenylindole (DAPI; 1:50.000; Molecular Probes, Leiden, Netherlands) during secondary antibody incubation. Secondary antibodies used were donkey anti-mouse DyLight (1:250; Jackson ImmunoResearch Laboratories Inc., West Grove, PA, United States) or donkey anti-goat Alexa Red (1:400; Jackson ImmunoResearch Laboratories Inc.). Conventional fluorescence microscopy was performed on a Nikon eclipse 80i light microscope equipped with a 40× objective (NA 0.75). Confocal images were obtained using a Zeiss Axiovert 200 M confocal microscope (Carl Zeiss Microscopy GmbH, Germany) equipped with a 63× water immersion objective (NA 1.2) plus 29 digital zoom. Excitation was performed with an air-cooled Argon ion laser (LASOS, RMC 7812Z, 488 nm) for GFP and a HeNE (LASOS, SAN 7450A, 543 nm) laser for DyLight.

### Western Blot

Cell lysis, western blot and subsequent analysis was performed as described earlier (Ji et al., 2017b). K_IR_2.1 antibody used was identical as used for immunofluorescence microscopy. Equal protein loading was determined by Ponceau staining.

### Patch-Clamp Electrophysiology

HEK293T cells were transfected with WT or ΔPEST K_IR_2.1 expression constructs together with a GFP expression construct to identify transfected cells. Inside-out patch clamp measurements on excised membrane patches were performed using an AxoPatch 200B amplifier controlled by pClamp9 software (Molecular Devices, Sunnyvale, CA, United States) at 21°C as described before (Ji et al., 2017b). To record K_IR_2.1 currents, inside-out patch-clamp measurements were performed using a ramp protocol ranging from −100 to +100 mV over 5 s from a holding potential of −40 mV. Bath solution contained (in mM): 125 KCl, 4 EDTA, 2.8 KH_2_PO_4_, 7.2 K_2_HPO_4_ (pH 7.2 with KOH), and pipette solution contained (mM): 145 KCl, 5 HEPES, 1 CaCl_2_ (pH 7.4 with KOH). Excised patches were placed in close proximity of the inflow region of the perfusion chamber. Measurements were started following washout of polyamines/Mg^2+^ from the channel pore, observed by the disappearance of current rectification.

Whole cell patch clamp measurements were done as described before (Houtman et al., 2012) using an AxoPatch 200B amplifier controlled by pClamp9 software at 21°C. Whole cell I_*KIR*__2.1_ measurements were performed by applying 1 s test pulses ranging between -120 and +30 mV, in 10 mV increments, from a holding potential of -40 mV, and with series resistance compensation of at least 70%. Signals were low-pass filtered at 2 kHz and sampled at 4 kHz. Liquid junction potential (LJP) was determined with the built in “Junction Potential Calculator” application of pCLAMP. Using the current solutions, LJP was 14.7 mV. Steady state current at the end of the pulse was normalized to cell capacitance and plotted versus test potential (corrected for LJP).

### Statistics

Group averages are presented as mean ± SEM, unless indicated otherwise. Differences between groups were tested by (un)paired Student’s *t*-test or two-way ANOVA followed by a *post hoc* Bonferroni test. Results with *P* < 0.05 were considered as statistically significant. Statistically analyses were performed using Prism 6 (GraphPad, CA, United States).

## Results

### Vertebrate K_IR_2.1 Proteins Contain a Conserved PEST-Domain

**FIGURE 1:**
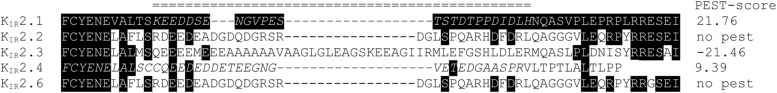
Amino acid alignment of C-termini of human K_IR_2.1, K_IR_2.2, K_IR_2.3, K_IR_2.4, and K_IR_2.6 encompassing the PEST domain region of K_IR_2.1 indicated by double line above the alignment. Amino acid sequences are depicted in single letter code. Identical residues with respect to K_IR_2.1 are depicted in white font on a black background. K_IR_2.4 contains a potential PEST sequence extending from 378 to 424 (KSSFPGSLTAFCYENELALSCCQEEDEDDETEEGNGVETEDGAASPR). PEST domains in K_IR_2.1 and K_IR_2.4 are indicated in italic. PEST scores are depicted at the right side of the sequences.

We aligned 31 K_I__R_2.1 amino acid sequences covering the phyla from fish to man (Houtman et al., 2014). Least sequence identity was observed between residues 380 and 415 in the C-terminal domain. However, since we noticed that this region was enriched in proline (P), glutamate (E), aspartate (D), serine (S), and threonine (T) residues, a hallmark of so-called PEST domains (Rechsteiner and Rogers, 1996), individual sequences were screened according to a PEST finding algorithm using the EMBOSS program epestfind. With a PEST score above 5, an amino-acid sequence will be considered as a genuine PEST domain. This revealed that all 31 sequences are characterized by a PEST domain having scores ranging between 8.7 (rainbow trout) and 24.5 (Opossum) with an average score of 19.4 (median 21.7) (Table 1). In addition, we added predicted K_IR_2.1 sequences of the lobe finned fish Coelacanth (XP_005992210) and of the primitive cartilaginous fish elephant shark (XP_007886827) whose sequences also contained PEST domains with a high PEST score (10.11 and 24.10, respectively) (Table 1). In contrast, no PEST domains were found in human K_IR_2.2, K_IR_2.3 or K_IR_2.6 channel proteins, while K_IR_2.4 contains a PEST domain (residues 378-424) with a PEST score of 9.39 that starts upstream from the K_IR_2.1 PEST domain (Figure 1).

**TABLE 1:** PEST scores of 33 vertebrate K_IR_2.1 protein sequences.

**Code**	**Scientific name**	**Common name**	**PEST sequence**	**Score**
Hs	*Homo sapiens*	Human	KEEDDSENGVPESTSTDTPPDIDLH	21.76
Pt	*Pan troglodytes*	Chimpanzee	KEEDDSENGVPESTSTDTPPDIDLH	21.76
MaMu	*Macaca mulatta*	Macaca	KEEDDSENGVPESTSTDTPPDIDLH	21.76
Eq	*Equus caballus*	Horse	KEEDDSENGVPESTSTDTPPDIDLH	21.76
Bt	*Bos taurus*	Bovine	KEEDDSENGVPESTSTDTPPDIDLH	21.76
Ss	*Sus scrofa*	Pig	KEEDDSENGVPESTSTDTPPDIDLH	21.76
Cf	*Canis familiaris*	Dog	KEEDDSENGVPESTSTDTPPDLDLH	21.95
Ua	*Ursus americanus*	American black bear	KEEDDSDNGVPESTSTDTPPDIDLH	21.60
Et	*Echinops telfairi*	Madagascar hedgehog	KEEDDSENGLPESTSTDTPPDMDLH	21.71
Oc	*Oryctolagus cuniculus*	European rabbit	KEEDDSENGVPESTSTDTPPDIDLH	21.76
Ml	*Myotis lucifugus*	Little brown bat	KEEDDSDNGVPESTSTDTPPDLDLH	21.78
Dn	*Dasypus novemcinctus*	Armadillo	KEEDDSENGVPESTSTDTPPDINLH	19.18
Mm	*Mus musculus*	Mouse	KEEEEDSENGVPESTSTDSPPGIDLH	21.07
Rn	*Rattus norvegicus*	Norwegian rat	KEEEDSENGVPESTSTDSPPGIDLH	19.51
St	*Spermophilus tridecemlineatus*	Thirteen-lined ground squirrel	KEEEDSENGVPESTSTDTPPDIDLH	21.92
Cp	*Cavia porcellus*	Guinea pig	KEEDDSENGVPESTSTDTPPDIDLH	21.76
Md	*Monodelphis domestica*	Opossum	KEEDDSENGLPESTSTDTPPDIDH	24.52
Oa	*Ornithorhynchus anatinus*	Platypus	HGVPESTSTDSPPDIDH	15.94
Gg	*Gallus gallus*	Chicken	KEEDEIDTGVPESTSTDTH	21.83
Cj	*Coturnix japonica*	Japanese quail	KEEDEIDTGVPESTSTDTH	21.83
Cl	*Columba livia*	Domestic pigeon	KEEDEIDTGVPESMSTDTH	17.21
Tg	*Taeniopygia guttata*	Zebra finch	KEEDEIDTGVPESMSTDTH	17.21
Tse	*Trachemys scripta elegans*	Red-eared Slider	KEEDESDNGVPESMSTDTLPDMDH	17.67
Lg	*Lampropeltis getula californiae*	California kingsnake	KEEEDSDNGVPESTSTDTH	24.28
Xt	*Xenopus tropicalis*	West-African clawed frog	KEEEGSDNGVPDSMSTDMH	11.36
Bb	*Blicca bjoerkna*	White bream	KEEGNGDSLGPGGTNTDTSSDSDH	16.26
Cc	*Cyprinus carpio*	Common carp	KEEGTGDSLGPGGTNTDTSSDSDH	18.38
Dr	*Danio rerio*	Zebrafish	KEEGHGDSLGPGGTNTETSSDSEH	14.34
Om	*Oncorhynchus mykiss*	Rainbow trout	KEETDEGNGGSVGPDVTH	8.70
Tr	*Takifugu rubripes*	Pufferfish	KEDTDEGNGGSVGPDGTQTDNISENEH	13.71
Ol	*Oryzias latipes*	Medaka	KEDMDEGNGSSVGPDGTQTDNISDTEH	13.55
Lc	*Latimeria chalumnae*	Coelacanth	KEEDDSDNGVPEIMSTDMH	10.11
Cm	*Callorhinchus milii*	Elephant shark	KDEEESEGGSPETVSAEAPPSTDH	24.10

*PEST scores were determined with aid of the epestfind webtool at http://mobyle.pasteur.fr/cgi-bin/portal.py$\Delta$form=epestfind.*

### The K_IR_2.1 PEST Domain Is Not Required for Normal Channel Protein Expression, Subcellular Localisation, Response to Chloroquine, or Rapid Protein Turnover Rate

**FIGURE 2:**
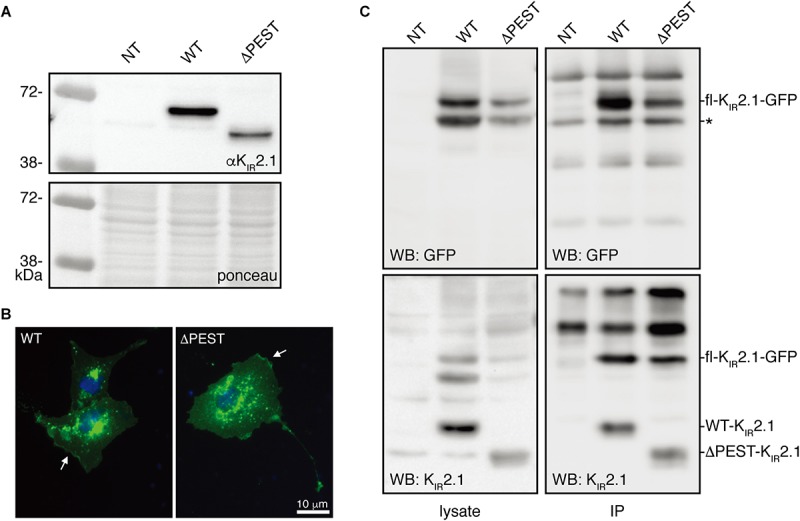
Expression analysis and channel formation of human WT and ΔPEST K_IR_2.1 protein. **(A)** Western blot depicting WT (approximately 50 kDa) and ΔPEST (approximately 47 kDa) K_IR_2.1 proteins expressed in HEK293T cells. Non-transfected cells (NT) were used as negative control. Ponceau staining depicts loading control. **(B)** Subcellular localization of ectopically expressed WT and ΔPEST K_IR_2.1 channel proteins in COS-7 cells. Arrows indicate membrane ruffles with K_IR_2.1 expression. **(C)** HEK293T cells were co-transfected with GFP-tagged murine K_IR_2.1 and either WT or ΔPEST K_IR_2.1. Non-transfected cells (NT) were used as negative control. K_IR_2.1-GFP was detected by GFP antibody (WB: GFP) for IP control, and N-terminal K_IR_2.1 antibody (WB: K_IR_2.1) was used to detect K_IR_2.1-GFP either WT or ΔPEST non-tagged K_IR_2.1 protein. Positions of K_IR_2.1-GFP, WT-K_IR_2.1 and ΔPEST-K_IR_2.1 are indicated. Lysate blots serve as immune-precipitation input control. ^*^IgG heavy chain.

A human K_IR_2.1 protein lacking the complete PEST domain (ΔPEST) was constructed to gain insight into the biological role of the PEST domain. Upon transfection in HEK293T cells, ΔPEST channel protein was detected on Western blot using an antibody against the N-terminus having, as expected, a lower apparent Mw as compared to WT channel proteins (Figure 2A). We next addressed the subcellular localization of ΔPEST K_IR_2.1 channel proteins upon ectopic expression in COS-7 cells. Twenty-four hour following transfection of cells with either WT or ΔPEST, immunostaining was performed using the N-terminal antibody against K_IR_2.1. Signals were found throughout the cells, but also in membrane ruffles indicative for plasma membrane localisation (Figure 2B).

To determine the potential of heterotetramerization, we co-transfected GFP-tagged WT K_IR_2.1 in HEK293T with either non-tagged WT or ΔPEST encoding construct and performed co-IP with GFP antibody. We were able to co-immunoprecipitate non-tagged WT, and also ΔPEST channel proteins, as detected using the N-terminal directed antibody for western blot (Figure 2C). Therefore, we conclude that the PEST domain is not required for interaction between K_IR_2.1 channel protein subunits.

**FIGURE 3:**
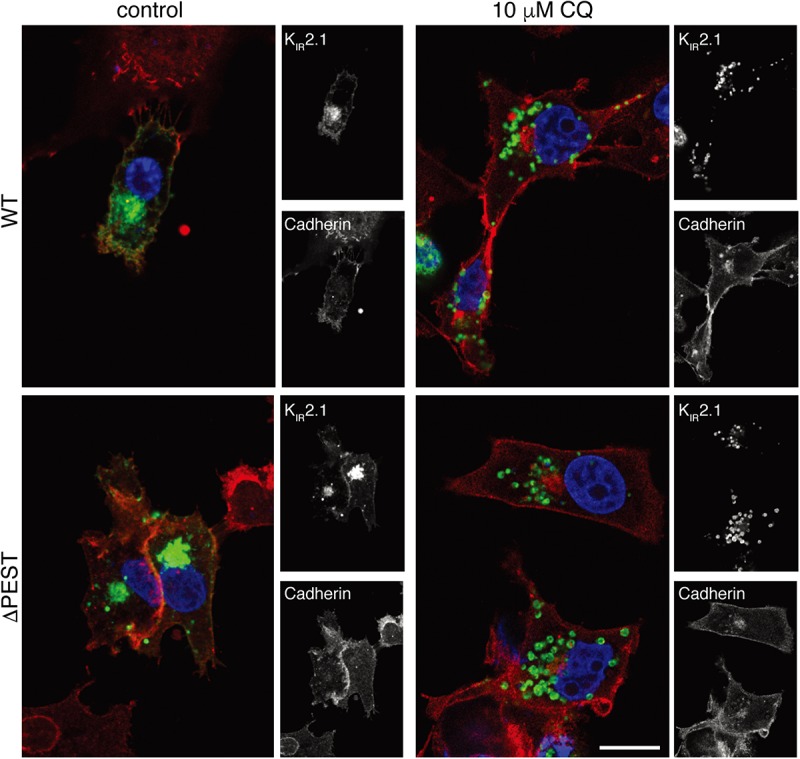
CQ treatment induces intracellular accumulation of WT and ΔPEST K_IR_2.1 protein in COS-7 cells. Confocal images of WT and ΔPEST K_IR_2.1 detected by N-terminal K_IR_2.1 antibody (green), and Cadherin (membrane staining) by Pan-Cadherin antibody (red). Single staining results are depicted on the right by b/w images. Scale bar indicates 10 μm.

K_IR_2.1 proteins are degraded by lysosomal degradation (Jansen et al., 2008; Varkevisser et al., 2013). Chloroquine application results in K_IR_2.1 accumulation upon chronic exposure (Jansen et al., 2008; Varkevisser et al., 2013). We next assessed the response of ΔPEST K_IR_2.1 protein to chloroquine exposure of 10 μM for 24 h in COS-7 cells by confocal microscopy. Both WT and ΔPEST K_IR_2.1 proteins displayed similar responses (Figure 3). Intracellular K_IR_2.1 accumulation was observed in what appeared as vesicle like structures, presumably lysosomes.

**FIGURE 4:**
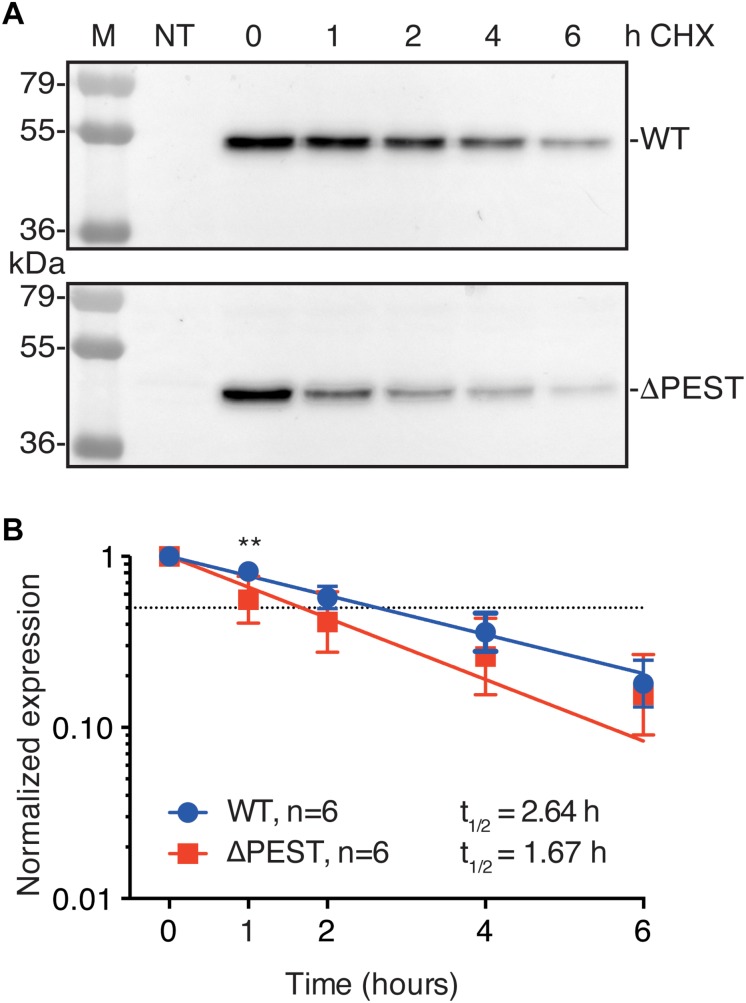
Cycloheximide (CHX) assay of K_IR_2.1 degradation in transfected HEK293T cells. **(A)** Example of WT and ΔPEST K_IR_2.1 protein degradation after exposure to 200 μg/mL CHX for different time intervals. Non-transfected (NT) cell were used as negative control. **(B)** Quantification of CHX assays to depict normalized K_IR_2.1 expression vs. timed CHX treatment. Dotted line indicates 50% of initial normalized K_IR_2.1 protein signal. ^∗∗^*P* < 0.01 WT vs. ΔPEST.

PEST domains have been associated in protein turnover rate, i.e., many short-lived proteins contain a PEST domain (Sandoval et al., 2006; Belizario et al., 2008; Meyer et al., 2011). Therefore, we tested protein turnover rates in transiently transfected HEK293T cells in the presence of 200 μg/mL CHX. WT and ΔPEST proteins displayed a time-dependent decrease in expression. Following 1 h of CHX treatment, a stronger decrease in ΔPEST expression compared to WT was found, however, no significant differences were detected on later time-points neither was there a significant difference in half life (T½ of 2.6 h vs. 1.7 h for the WT and ΔPEST K_IR_2.1 protein, respectively) (Figure 4). Thus, removing the PEST domain from the K_IR_2.1 protein does not decrease protein turnover rate.

### Human K_IR_2.1 ΔPEST Channels Produce Typical Inward Rectifying Potassium Currents With Enhanced Rectification

**FIGURE 5:**
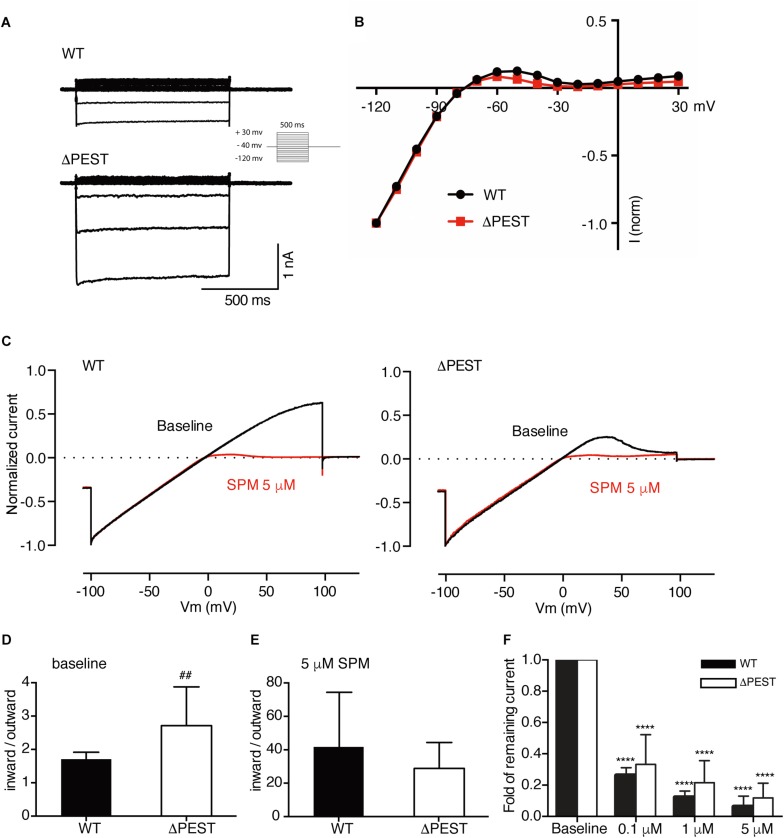
Electrophysiological analysis of human WT and ΔPEST K_IR_2.1 channels transiently transfected in HEK293T cells. **(A)** Representative current traces of WT and ΔPEST I_*KIR2.1*_ recorded in whole cell mode. **(B)** Normalized current–voltage relation curve of WT and ΔPEST I_*KIR2.1*_ (mean ± SEM; WT *n* = 18, ΔPEST *n* = 18), note that error bars are smaller than symbols at each point. **(C)** Steady state I_*KIR2.1*_ traces from WT and ΔPEST channel containing excised inside–out patches elicited by a voltage ramp protocol from -100 to + 100 mV over 5 s, under baseline conditions (black) and upon application of 5 μM spermine (red). **(D,E)** Quantification of rectification index (inward current at –80 mV divided by outward current at +50 mV) of WT and ΔPEST I_*KIR2.1*_ from ramp protocol elicited currents in inside-out mode without (**D**, baseline) and in the presence of 5 μM spermine **(E)** (mean ± SD, WT *n* = 10, ΔPEST *n* = 10). **(F)** Quantification of normalized outward current (at +50 mV) from WT and ΔPEST channels in inside-out patch clamp under baseline conditions and with increasing spermine concentrations. ${}^{\#\,\#}$*P* < 0.01 vs. WT; ^****^P < 0.0001 vs. baseline (mean ± SD, WT *n* = 10, ΔPEST *n* = 10).

We assessed inward rectifier current formation of WT and ΔPEST channels by whole cell patch clamp electrophysiology on transiently transfected HEK293T cells. Both channel types resulted in the formation of typical inwardly rectifying potassium currents and corresponding IV curves (Figure 5A). Comparison of rectification (maximal outward current vs. maximal inward current) indicated no statistical difference in rectification between WT and ΔPEST channels in the whole cell mode (at −60 mV, 0.119 ± 0.022 vs. 0.085 ± 0.014 (*P* = 0.31) for WT and ΔPEST, respectively) (Figure 5B).

To better assess inward rectification properties, inside-out measurements of WT and ΔPEST K_IR_2.1 channels were performed in the absence of polyamines and Mg^2+^ using a ramp protocol from −100 to +100 mV (Figure 5C). Under baseline conditions almost straight voltage-current relationships were observed between −100 and +50 mV. Between +50 and +100 mV some rectification was observed for WT channels. In contrast, ΔPEST K_IR_2.1 channels produced more pronounced rectification between +40 and +100 mV (Figure 5C). Quantification demonstrated a significantly stronger rectification (inward at −80 mV/outward at +50 mV) for ΔPEST compared to WT K_IR_2.1 channel (2.7 ± 1.2 vs. 1.7 ± 0.2, *P* < 0.01, *n* = 10, mean ± SD) (Figure 5D). Upon application of 5 μM spermine, both types of channels displayed strong rectification (28.8 ± 15.6 vs. 41.7 ± 32.6; n.s. for ΔPEST and WT currents) (Figure 5E). Finally, we observed a similar dose-dependent decrease in remaining current at +50 mV upon perfusion with 0.1, 1 and 5 μM spermine, respectively (Figure 5F) (WT: baseline vs. 0.1 μM: *P* < 0.0001, 0.1 μM vs. 1 μM and 5 μM: *P* < 0.0001, 1 μM vs. 5 μM: *P* < 0.05; ΔPEST: baseline vs. 0.1 μM: *P* < 0.0001, 0.1 μM vs. 1 μM and 5 μM: *P* < 0.05 and *P* < 0.0001, respectively, 1 μM vs. 5 μM: n.s.) The strongest decrease in current was observed upon perfusion with 0.1 μM spermine (0.26 ± 0.05 and 0.33 ± 0.19 fold for WT and ΔPEST K_IR_2.1 current, respectively).

### Snake ΔPEST K_IR_2.1 Channels

**FIGURE 6:**
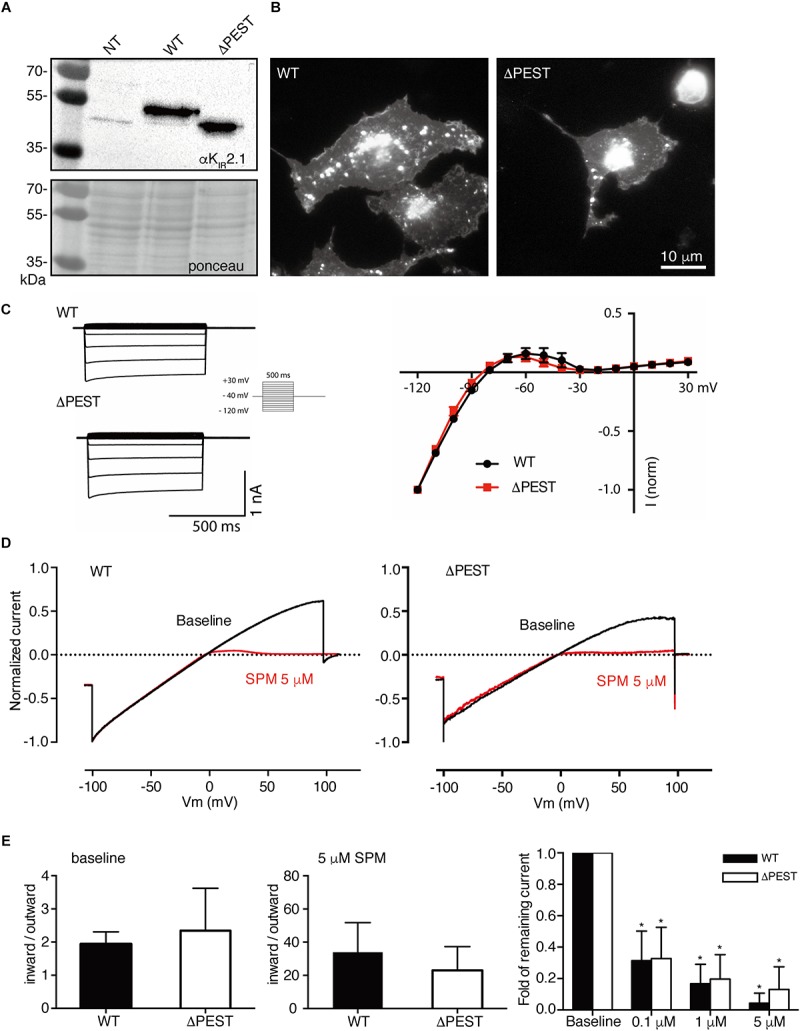
Expression analysis and channel formation of snake WT and ΔPEST K_IR_2.1 protein and electrophysiological analysis of formed channels in transiently transfected HEK293T cells and COS-7 cells. **(A)** Western blot depicting WT (approximately 50 kDa) and ΔPEST (approximately 47 kDa) K_IR_2.1 protein. Non-transfected cells (NT) were used as negative control. Ponceau staining depicts loading control. **(B)** Subcellular localization of ectopically expressed WT and ΔPEST K_IR_2.1 channel proteins in COS-7 cells. Apart from plasma membrane staining, intracellular aggregates were observed. **(C)** Representative current traces of WT and ΔPEST I_*KIR2.1*_ recorded in whole cell mode (left) and normalized current–voltage relation curves of WT and ΔPEST I_*KIR2.1*_ (right) (mean ± SEM, WT *n* = 9, ΔPEST *n* = 7). **(D)** Steady state I_*KIR2.1*_ traces from WT and ΔPEST channel containing inside–out patches elicited by a voltage ramp protocol from -100 to + 100 mV, under baseline conditions (black) and upon application of 5 μM spermine (red). **(E)** Quantification of rectification index (inward current at –80 mV divided by outward current at +50 mV) of WT and ΔPEST I_*KIR2.1*_ from ramp protocol elicited currents in inside-out mode without (left panel) and in the presence of 5 μM spermine (middle panel) (mean ± SD, WT *n* = 11, ΔPEST *n* = 24). Quantification of normalized outward current (at +50 mV) from WT and ΔPEST channels in inside-out patches under baseline conditions and with increasing spermine concentrations (right panel). ^*^P < 0.05 vs. baseline (mean ± SD, WT *n* = 10, ΔPEST *n* = 10).

Given the high level of conservation of the PEST domain across the vertebrate phyla, we hypothesized that enhanced rectification in ΔPEST channels could also be observed in the previously cloned snake K_IR_2.1 channel (Houtman et al., 2014). For this purpose, a snake ΔPEST K_IR_2.1 was generated similarly, as its human counterpart. Figures 6A,B depicts expression of snake WT and ΔPEST channels in HEK293T cells (Figure 6A) and COS-7 cells (Figure 6B) by Western blot and immunofluorescence microscopy, respectively.

Both WT and ΔPEST channels from snake produced typical K_IR_2.1 currents as demonstrated by whole cell patch clamp electrophysiology (Figure 6C). When using inside-out patch clamp measurements in the absence of polyamines and Mg^2+^ no statistical difference in rectification index was observed (1.9 ± 0.4 vs. 2.4 ± 1.3; *P* = 0.13 for WT and ΔPEST, respectively, mean ± SD) (Figures 6D,E). Distribution analysis of rectification index of each patch measured, demonstrated a larger variation and rightward shift in ΔPEST channels compared to WT channels, although not as prominent as found for the human variants (Supplementary Figure S1). As for the human channels, application of spermine dose-dependently enhanced rectification (Figure 6E) (WT: baseline vs. 0.1 μM: *P* < 0.05, 0.1 μM vs. 1 μM and 5 μM: *P* < 0.05 and *P* < 0.0001, respectively, 1 μM vs. 5 μM: n.s.; ΔPEST: baseline vs. 0.1 μM: *P* < 0.05, 0.1 μM vs. 1 μM and 5 μM: *P* < 0.01 and *P* < 0.0001, respectively, 1 μM vs. 5 μM: n.s.).

## Discussion

In the current work we established the existence of a conserved PEST domain in the C-terminus of the K_IR_2.1 potassium ion channel protein. The PEST domain is not essential for normal plasma membrane expression of K_IR_2.1 protein, tetramerization with wildtype channel proteins, intracellular K_IR_2.1 accumulation in response to chronic chloroquine treatment or rapid protein degradation. However, deletion of the PEST domain increases rectification behavior of the human K_IR_2.1 channels.

PEST domains are defined by a specific signature, i.e., a stretch of amino acids rich in P, E, D, S and T most often confined by positively charged residues on both sides, rather than by a determined sequence motif. This may explain why this domain has not been recognized in the K_IR_2.1 protein before. Following the identification of PEST domains, the notification of the presence of PEST domains in many short living proteins stood at the basis of the PEST hypothesis, stating that PEST domains destabilize the protein in which they are present (Rogers et al., 1986). However, the identification of PEST domains in long-living proteins did not favor the PEST hypothesis, neither did the observations that deleting a PEST domain did not necessarily increase half-life (e.g., Pakdel et al., 1993; Xiao et al., 2014). Upon ectopic expression in HEK293 cells, K_IR_2.1 proteins have a short half-life (2.64 h). Deletion of the PEST domain did not increase T½ which is in contrast to the original PEST domain hypothesis as mentioned above. From these results we conclude that the PEST domain in K_IR_2.1 proteins does not promote protein instability and is not responsible for rapid protein degradation.

The human K_IR_2.1 PEST domain (residues 385–409) is located between the ER export signal FCYENE (374–379) and the PDZ binding domain ESEI (425–427). Similarly, the snake K_IR_2.1 PEST (383–401) is located between ER export signal (372–377) and PDZ binding domain (422–425). In crystallization studies, the last 57 residues of the mouse K_IR_2.1 channels were found to lack intrinsic structural rigidity, and it was suggested that this domain might require interactions with other regions of the protein and/or cytoplasmic proteins to adopt one or more defined conformations (Pegan et al., 2005). Therefore, the proline-rich PEST domain by itself might form a flexible linker domain between the two sequence conserved domains, and might allow for protein–protein interactions without affecting other structural domains of the channel. We can speculate that this would allow interaction of the PDZ binding domain with a range of different proteins depending on the cell type in which the channel is expressed. If so, this will provide versatility to this channel which is expressed in many different cell types and tissues (De Boer et al., 2010). The question then remains however, why the K_IR_2.2, K_IR_2.3, and K_IR_2.6 channel proteins do not contain a PEST domain between its ER export and PDZ domains. Furthermore, it does not explain evolutionary conservation of the PEST motif if only a flexible linker in this region of the K_IR_2.1 channel would serve the same purpose. On the other hand, domain linker regions may also serve an important function in the interplay between different domains (Gokhale and Khosla, 2000).

Inward rectification in K_IR_2.1 channels depends on polyamines entering the channel from the cytosolic side. Enormous progress in the understanding of the mechanism has been obtained but knowledge of all mechanisms involved at the molecular level is far from complete and consensus has not been reached (Nichols and Lee, 2018). As rectification at strong positive potentials is stronger in ΔPEST channels than in WT using inside-out patches following spermine washout, enhanced rectification appears an intrinsic property of the PEST domain lacking channels. Nevertheless, upon spermine application, strong rectification ensues in ΔPEST channels demonstrating that the basic mechanism of (bulk) rectification is not affected. We can only speculate on the mechanism of stronger rectification. Deletion of the PEST domain may have a charge effect on the protein that results in altered structural adaptations upon depolarization and thus induce subtle effects on rectification. Furthermore, the deletion may affect interactions with, not yet identified, cellular constituents at this site that play a role in rectification. Rectification effects in the snake K_IR_2.1 channel upon PEST deletion are less prominent, which might be related to the reduced length of the PEST domain in this species. However, the size of the PEST domain seems unrelated to the evolutionary pathways followed. In the same phylum PEST domains have different lengths (e.g., rainbow trout, 18 residues vs. white bream, 24; California kingsnake, 19 vs. red-eared slider, 24).

A potential physiological role for the PEST domain in K_IR_2.1 channels awaits further work, in which *in vivo* models with ubiquitous expression of ΔPEST channels may provide first clues into which of the many cell types that express K_IR_2.1 channel proteins, the PEST domain plays a prominent role. Only then can clinical implications be envisioned.

## Data Availability

The datasets generated for this study are available on request to the corresponding author.

## Author Contributions

MQ, YJ, MH, MV, FR, BK, and MvdH performed the research. MQ, YJ, MH, MV, FR, and MvdH analyzed the results. MvdH designed the study. MQ and MvdH wrote the manuscript. All authors reviewed the final version of the manuscript.

## Conflict of Interest Statement

The authors declare that the research was conducted in the absence of any commercial or financial relationships that could be construed as a potential conflict of interest.

## Footnotes

1 http://emboss.bioinformatics.nl/cgi-bin/emboss/epestfind, assessed on April 6, 2018

## Supplementary Material

FIGURE S1 Distribution analysis of rectification classes of individual cell patches from human (left panel) and snake (right panel) WT and ΔPEST channel currents measured in the inside-out mode. Rectification indexes (inward current at −80 mV divided by outward current at +50 mV) from individual measurements were categorized in fifteen equal discrete classes from 0.0 to 8.0 The percentage of cell patches belonging to an individual class (all patches = 100%) are indicated on the y-axis. Whereas in most cell inside-out patches containing human WT K_IR_2.1, rectification indexes were found to be between 1.0 and 2.0, all human ΔPEST patches displayed rectification indexes of 1.5 and higher. In snake K_IR_2.1 WT and ΔPEST patches, the distribution of rectification classes displayed more overlap. Distribution analysis quantified from measurements depicted as mean values in Figures 5D,E and 6E.

## Abbreviations

Δ PEST: K_IR_2.1 protein lacking the complete PEST domain
CHX: cycloheximide
CQ: chloroquine
PEI: polyethylenimine
SPM: spermine
SUMO: small ubiquitin like modifier.
