# Supplementary figures and images for: Identification of a PEST Sequence in Vertebrate KIR2.1 That Modifies Rectification
Source: Front Physiol. 2019 Jul 5;10:863. doi: 10.3389/fphys.2019.00863 (PMC6624654; doi:10.3389/fphys.2019.00863)

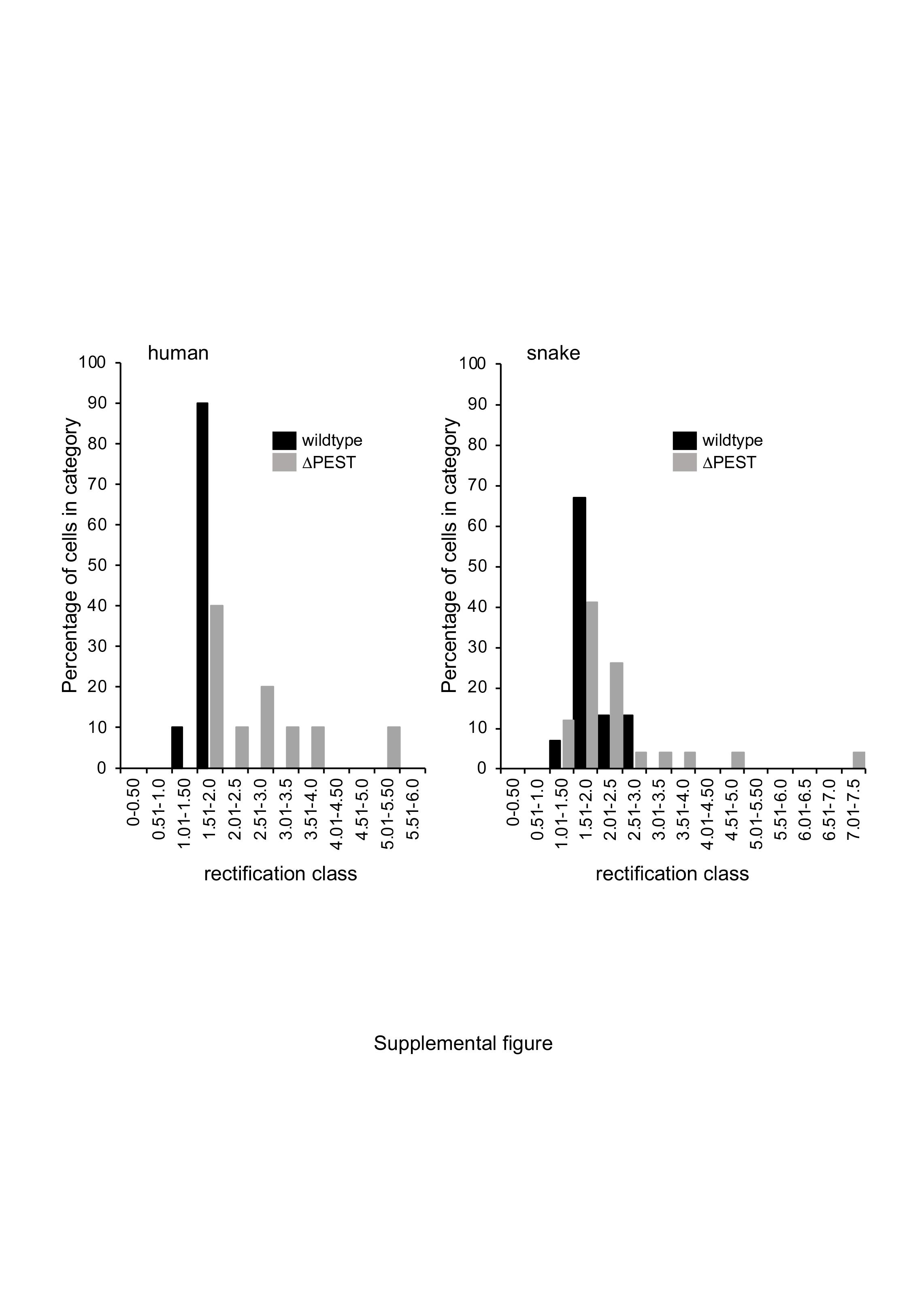

Supplement: FIGURE S1 — Distribution analysis of rectification classes of individual cell patches from human (left panel) and snake (right panel) WT and ΔPEST channel currents measured in the inside-out mode. Rectification indexes (inward current at −80 mV divided by outward current at +50 mV) from individual measurements were categorized in fifteen equal discrete classes from 0.0 to 8.0 The percentage of cell patches belonging to an individual class (all patches = 100%) are indicated on the y-axis. Whereas in most cell inside-out patches containing human WT KIR2.1, rectification indexes were found to be between 1.0 and 2.0, all human ΔPEST patches displayed rectification indexes of 1.5 and higher. In snake KIR2.1 WT and ΔPEST patches, the distribution of rectification classes displayed more overlap. Distribution analysis quantified from measurements depicted as mean values in Figures 5D,E and 6E. [file Image_1.JPEG]
